# Nuclear accumulation of polyglutamine disease proteins and neuropathology

**DOI:** 10.1186/1756-6606-2-21

**Published:** 2009-07-03

**Authors:** Lauren S Havel, Shihua Li, Xiao-Jiang Li

**Affiliations:** 1 Department of Human Genetics, Emory University School of Medicine, Atlanta, GA, 30322, USA

## Abstract

There are nine inherited neurodegenerative disorders caused by polyglutamine (polyQ) expansion in various disease proteins. Although these polyglutamine proteins have different functions and are localized in different subcellular regions, all the polyQ diseases share a common pathological feature: the nuclear accumulation of polyQ disease proteins and the formation of inclusions. The nuclear accumulation of polyQ proteins in turn leads to gene transcriptional dysregulation and neuropathology. Here we will discuss potential mechanisms behind the nuclear accumulation of mutant polyQ proteins, since an understanding of how polyQ proteins accumulate in the nucleus could help elucidate the pathogenesis of these diseases and develop their treatment.

## 

There are nine inherited neurodegenerative disorders, including Huntington's disease (HD), dentatorubral-pallidoluysian atrophy (DRPLA), spinal bulbar muscular atrophy (SBMA), and the spinocerebellar ataxias (SCA) 1,2,3,6,7 and 17, which are caused by a polyglutamine (polyQ) expansion in their respective disease proteins [1]. The polyQ domain is encoded by polymorphic CAG repeats that are expanded in polyQ diseases. For example, in Huntington's disease the polyQ domain is in the N-terminal region of the HD protein, huntingtin (htt), and its expansion to more than 37 glutamines leads to the neurological symptoms of HD. All the polyglutamine disorders share several common pathological features, including the nuclear accumulation and aggregation of the disease proteins. Neuronal nuclear inclusions are considered to be a histopathological hallmark of the polyQ diseases and are even observed in disease brains in which normal polyQ proteins are predominantly expressed in the cytoplasm. Although the role of nuclear inclusions in pathology is not fully understood, what is clear is that the inclusions result from the nuclear accumulation of polyQ-expanded proteins. Mutant polyQ proteins in the nucleus can abnormally interact with nuclear proteins, such as transcription factors, leading to transcriptional dysregulation [2]. The preferential accumulation of mutant polyQ proteins in neuronal nuclei may be associated with the selective neuropathology seen in polyQ diseases. Thus, it is important to understand how polyQ expansions can cause the accumulation of polyQ proteins in neuronal nuclei. Such an understanding would tell us much about the selective neuropathology of polyQ diseases and also help us develop effective therapeutics for these diseases. In this review, we will discuss the potential mechanisms underlying the accumulation of polyQ-expanded proteins in neuronal nuclei.

## Nuclear accumulation of mutant polyglutamine proteins

In all polyQ diseases, the disease proteins are ubiquitously expressed; however, cell loss is restricted to the brain cells of polyQ disease patients. The context of the polyQ proteins and their interacting proteins may determine the selective neuronal loss seen in distinct brain regions in the different polyQ diseases (Table 1). Also, the selective neuropathology appears to be associated with the preferential accumulation of expanded polyQ proteins in neuronal cells, as the presence of nuclear polyQ proteins is evident in all polyQ disease brains. A prime example of this is that htt, which is normally distributed in the cytoplasm, can accumulate in the nucleus when its polyQ tract is expanded. Immunohistochemical data from the brains of HD patients reveal the presence of nuclear htt inclusions in the affected brain regions of both juvenile and adult patients [3,4]. Patients with other polyQ diseases, such as SCA1, SCA3, SCA7, SCA17, DPRLA, and SBMA, also show nuclear polyQ inclusions in the affected brain regions [1]. Even in the brains of patients with SCA2 and SCA6, which are caused by a polyQ expansion in the cytoplasmic proteins ataxin-2 and ataxin-6, respectively, there is evidence for the presence of polyQ inclusions in the nuclei of neuronal cells [5,6]. Moreover, linking an expanded polyQ repeat to the cytoplasmic protein Hprt results in the formation of nuclear polyQ inclusions in the brains of transgenic mice [7]. Thus, despite different subcellular localizations of the normal polyQ proteins, mutant proteins with their expanded polyQ repeats commonly form nuclear inclusions or accumulate in the nucleus; such a common feature could be associated with the selective neuropathology of polyQ diseases.

**Table 1:** A summary of the nine inherited polyglutamine repeat disorders.

**Disease**	**Disease protein**	**Normal subcellular localization**	**Affected brain regions**
Huntington's disease (HD)	Huntingtin (htt)	Cytoplasm	Striatum and cortex

Spinocerebellar ataxia 1 (SCA1)	Ataxin-1	Nuclear and cytoplasmic	Cerebellum

Spinocerebellar ataxia 2 (SCA2)	Ataxin-2	Cytoplasmic	Cerebellar Purkinje cells

Spinocerebellar ataxia 3 (SCA3)	Ataxin-3	Nuclear and cytoplasmic	Ventral pons and substantia nigra

Dentatorubral-pallidoluysian atrophy (DRPLA)	Atrophin-1	Nuclear and cytoplasmic	Cerebral cortex

Spinocerebellar ataxia 6 (SCA6)	Ataxin-6	Membrane associated	Cerebellar Purkinje cells

Spinocerebellar ataxia 7 (SCA7)	Ataxin-7	Nuclear and cytoplasmic	Cerebellar Purkinje cells, brain stem, spinal cord

Spinal and bulbar muscular atrophy (SBMA)	Androgen receptor (AR)	Nuclear and cytoplasmic	Motor neurons

Spinocerebellar ataxia 17 (SCA17)	TBP	Nuclear	Cerebellar Purkinje cells

Included are the polyQ proteins, their normal subcellular localization, and affected brain regions.

PolyQ inclusions in the nucleus are colocalized with ubiquitin, proteasome components, and heat shock proteins [3,8-10]. These findings suggest that polyQ protein deposits are targeted by cellular clearing systems. PolyQ inclusions are likely to be compact structures consisting primarily of the polyQ protein itself, since expanded polyQ repeats can cause self-association of polyQ peptides, leading to various forms of the proteins with different conformations [11]. Examination of the brains of HD patients indicates that only truncated N-terminal htt fragments with an expanded polyQ tract are capable of forming nuclear inclusions, as these nuclear inclusions can only be labeled by antibodies against the N-terminal, but not the internal or C-terminal, region of htt [3,4]. Western blot analysis of HD mouse models that express full-length mutant htt reveals the presence of a number of N-terminal fragments of various sizes [12-14]. Cellular models of HD have revealed a number of htt fragments containing the polyQ tract and various proteolytic cleavage sites, including those for caspase-3, caspase-6, and calpains [15-19]. Nonetheless, which fragments can accumulate in the nucleus and how they contribute to neuropathology remain to be investigated. Despite these unanswered questions, we know that the presence of N-terminal htt fragments in HD mouse brains can be detected as early as two months prior to the obvious neurological phenotype, which does not appear until the age of four to five months, indicating that the generation and accumulation of N-terminal htt precede neurological symptoms [13].

The fact that small htt fragments form nuclear inclusions suggests that a shorter peptide with a larger polyQ tract tends to misfold and aggregate more rapidly. In other polyQ diseases, it is also evident that shorter polyQ proteins are prone to misfolding and aggregation. For example, western blot analysis of a transgenic mouse model of DRPLA showed the presence of a small N-terminal fragment of atrophin-1 [20,21]. Similarly, brain samples from SCA3 patients as well as mice transgenic for full-length ataxin-3 with 71Q showed the production of a C-terminal truncated fragment with the expanded polyQ domain [22]. Furthermore, the production of small polyQ protein fragments is found to be required for aggregation [23], indicating that proteolytic processing of polyQ proteins is critical for the generation of toxic and misfolded polyQ proteins.

Although the role of nuclear inclusions remains controversial, the formation of these nuclear inclusions clearly results from the nuclear accumulation of misfolded and toxic forms of mutant polyQ proteins. The toxicity of small N-terminal htt fragments with an expanded polyQ repeat is evidenced by the severe neuropathological phenotypes of transgenic mice expressing truncated and polyQ-expanded htt. For example, the ubiquitous expression of exon 1 of mutant htt in the transgenic R6/2 model of HD is sufficient to produce a progressive and severe neurological phenotype. These mice exhibit the abundant nuclear inclusions, motor abnormalities, weight loss, and brain atrophy indicative of early neurodegeneration [24,25]. The neuronal toxicity of mutant htt can be enhanced by its nuclear accumulation, as the addition of a nuclear localization sequence (NLS) to exon 1 of mutant htt increases toxicity in neuroblastoma cells [26] and also results in an accelerated neurological phenotype in transgenic mice [27].

## Nuclear effects of mutant polyQ proteins

When localized to the nucleus, polyQ-expanded proteins aberrantly interact with a variety of transcription factors, many of which contain a polyQ or glutamine-rich domain. Certain transcription pathways, including those involving the cAMP response element (CRE)-binding protein (CREB) [28,29], Sp1 [30,31], and PGC-1alpha [32], have been implicated in the pathogenesis of multiple polyQ diseases. Soluble mutant htt seems to be able to abnormally bind transcription factors to affect their transcriptional activity [30,31]. In SCA17 mouse brains, aggregated polyQ proteins could also sequester the transcription factor TF-IIB [33], though it has been reported that there is not a direct correlation between the presence of nuclear polyQ inclusions and neurodegeneration in other polyQ disease models [34-36]. It seems that protein context determines specific protein interactions and their consequences in polyQ diseases.

It is evident that mutant polyQ proteins can affect transcriptional activities [37]. Microarray experiments using brain mRNAs from various polyQ mouse models have revealed some overlap in the expression changes induced by the different polyQ disease proteins. For example, comparing gene expression profiles of HD mouse models that express exon 1 mutant htt (R6/2) and full-length mutant htt shows no discernable differences between the full-length and fragment models, despite the delayed changes in full-length htt mouse brains, suggesting that N-terminal fragments of mutant htt are the major pathogenic form to induce altered gene transcription [38]. Although it is expected that mutant polyQ proteins in the nucleus can affect gene expression, whether and how transcriptional dysregulation can lead to neuronal dysfunction or cell death in the brain is not entirely clear.

## Preferential accumulation of polyQ-expanded proteins in the nucleus

Although immunocytochemistry studies show that some normal htt can localize to the nucleus [39], nuclear fractionation of HD mouse brains clearly indicates that the majority of full-length mutant htt is cytoplasmic and that smaller N-terminal htt fragments are enriched in the nucleus [13,14,40]. Understanding how a polyQ protein that is normally distributed in the cytoplasm can accumulate in the nucleus when its polyQ tract is expanded is critical for gaining insight into the pathogenic mechanisms of polyQ repeat disorders. This is especially important for understanding the pathogenesis of HD, as N-terminal htt does not carry the conserved nuclear import sequences.

Several putative nuclear localization signals have been found in htt [41]; however, they are not localized in N-terminal htt fragments that are able to accumulate in the nucleus. Because only small N-terminal htt fragments are able to accumulate in the nucleus, the belief is that these htt fragments enter the nucleus via a passive diffusion mechanism. We know that proteins <40 kDa can diffuse freely through the nuclear pore, whereas proteins >40 kDa normally rely on active transport [42]. N-terminal htt fragments localize to the nucleus, while the large fragments (>60 kDa) showed perinuclear and cytoplasmic but no nuclear localization [43], suggesting that smaller fragments are prone to passive diffusion. Indeed, the transgenic mouse model of HD expressing the short exon 1 or N171 fragment of mutant htt consistently showed more abundant nuclear aggregates and a more severe neurological phenotype than HD mice expressing full-length mutant htt [2]. The delayed nuclear accumulation of mutant htt and the late onset of neurological phenotypes in HD knock-in mice are consistent with a time-dependent accumulation of N-terminal htt fragments.

If small polyQ proteins can be freely translocated between the nucleus and cytoplasm, why do they preferentially accumulate in the nucleus to form nuclear inclusions? Cornett *et al *have demonstrated that a polyQ expansion can prevent mutant htt from being exported from the nucleus. The presence of an expanded polyQ tract reduces the association of N-terminal htt with the translocated promoter region protein (Tpr) [44]. Tpr is a nuclear pore protein that localizes to the nucleoplasmic side of the nuclear pore complex and exports molecules from the nucleus [45-47]. Expanded htt exhibits decreased interaction with Tpr compared with wild-type htt and thereby shows reduced nuclear export and increased nuclear accumulation [44]. Thus their study suggests that polyQ-expanded htt is prone to misfolding in the nucleus, which subsequently reduces its ability to exit the nucleus. This study also raises the interesting issue of whether the nuclear environment itself favors the misfolding of polyQ proteins.

## Regulation of the nuclear accumulation of polyQ proteins

Since polyQ expansions cause proteins to misfold and aggregate, clearing misfolded polyQ proteins is crucial to prevent their accumulation. Protein degradation via the ubiquitin-proteasome system (UPS) and autophagy are the major mechanisms to remove polyQ proteins in the cytoplasm. Because autophagy is not seen in the nucleus, it is the nuclear UPS that plays a major role in clearing mutant polyQ proteins in the nucleus. *In vitro *experiments using cultured cells have shown that overexpressed polyQ proteins can impair the function of the UPS [48-50]; however, the real question is whether this impairment occurs in the brains of mouse models expressing transgenic mutant polyQ proteins. Several groups using different mouse models of polyQ diseases have found no decrease in UPS activity in the brain tissues of mutant mice [51-55]. Hence the accumulation of mutant polyQ proteins in the nucleus is likely due to an intrinsic difference in the neuronal nuclear UPS activity. One important issue in this regard is whether the nuclear UPS has a lower activity than the cytoplasmic UPS, such that the nuclear UPS cannot efficiently degrade polyQ proteins, leading to the preferential accumulation of mutant polyQ proteins in the nucleus. Using fractionation and biochemical assays of the UPS activity, Zhou *et al *demonstrated that nuclear UPS activity is indeed lower than in the cytoplasm [13]. The difference between nuclear and cytoplasmic UPS activities was also demonstrated by targeting a fluorescent UPS reporter to the cytoplasm and nucleus, which again shows that UPS activity is lower in the nucleus than in the cytoplasm [55].

Another relevant question is why mutant polyQ proteins accumulate and form inclusions in the nucleus in an age-dependent manner. Aging is reported to increase cellular oxidative stress, which can damage the UPS and may cause an age-dependent decline in UPS activity [56,57]. Biochemical and fluorescent UPS reporter assays have in fact revealed an age-dependent decline in mouse brain UPS activity. Moreover, this decline is correlated with the observed age-dependent increase in nuclear htt accumulation and aggregation [13,55]. Further buttressing this correlation, increased nuclear accumulation of transfected mutant htt was found in cultured cells that were treated with proteasome inhibitors [13,44,50]. Thus, an age-dependent decrease in the clearance of misfolded polyQ proteins explains the late onset of nuclear polyQ protein accumulation and the associated neurological phenotypes.

Heat shock proteins are molecular chaperones that recognize and refold misfolded proteins, such as polyQ protein fragments, and the expression of endogenous chaperones, such as Hsp70, is decreased in mouse models of polyQ diseases [58,59]. Conversely, overexpression of heat shock proteins decreases the half-life of mutant polyQ proteins expressed in cell culture [60,61]. Although we have yet to establish whether Hsp activity is reduced in the nucleus by aging or mutant polyQ proteins, it is likely that enhancing nuclear Hsp activity or increasing the clearance of nuclear mutant polyQ proteins via the UPS should decrease the nuclear accumulation of polyQ proteins and ameliorate polyQ-mediated neuropathology.

As discussed above, protein-protein interactions can regulate the nuclear accumulation of polyQ proteins. In addition, posttranslational modifications are important for the nuclear accumulation of polyQ proteins, as well. A good example of this is that phosphorylation of the S776 residue in ataxin-1 can enhance its nuclear accumulation [62]. Furthermore, the first 17 amino acids of htt are found to be important for its nuclear localization [63]. Given that these N-terminal amino acids are conserved in different species, we need to explore whether their phosphorylation and other modifications can influence the nuclear accumulation of mutant htt.

## Concluding remarks

The nuclear accumulation of toxic polyQ proteins is necessary for the nuclear toxic effects of polyQ proteins. The fact that nuclear polyQ inclusions are a pathological hallmark of polyQ diseases indicates that the expanded polyQ tract can cause various proteins to accumulate in the nucleus. Moreover, the age-dependent decrease in nuclear UPS activity may account for the age-dependent accumulation of polyQ proteins in the nucleus. The intrinsically low UPS activity in the neuronal nuclei may contribute to the preferential accumulation of mutant polyQ proteins in neuronal nuclei. For those polyQ proteins that are normally present in the cytoplasm, proteolytic processing of these proteins to generate small truncated proteins that contain an expanded polyQ repeat and are able to enter the nucleus via a passive diffusion mechanism is important for their nuclear accumulation. In addition, protein interactions and posttranslational modifications, such as phosphorylation and acetylation, also affect the nuclear accumulation of polyQ proteins. In the case of HD, we have yet to determine which N-terminal htt fragments are generated and enter the neuronal nuclei in the brain and whether posttranslational modifications influence their nuclear accumulation.

Of course the ultimate goal in studying polyQ diseases is to determine the best targets for therapeutics to treat them. The model presented in this review suggests several such possible targets. First, inhibition of the cleavage of toxic fragments from the full-length htt may prevent its nuclear accumulation and nuclear dysfunction. Second, improving the function of nuclear clearing systems, such as the UPS and chaperones, could reduce the nuclear accumulation of mutant polyQ proteins. Third, preventing or reducing the aberrant interactions between the soluble polyQ proteins and transcription factors should also reduce polyQ-induced toxic effects. Thus, understanding the mechanism underlying the nuclear accumulation of mutant polyQ proteins could help us develop effective therapies for polyQ diseases.

## Competing interests

The authors declare that they have no competing interests.

## Authors' contributions

LSH, SHL, and XJL wrote the manuscript. All authors read and approved the final manuscript.
